# Supplementary material for: Identification and functional analysis of non-coding regulatory small RNA FenSr3 in Bacillus amyloliquefaciens LPB-18
Source: PeerJ. 2023 May 15;11:e15236. doi: 10.7717/peerj.15236 (PMC10194069; doi:10.7717/peerj.15236)
Supplement: Supplemental Information 4 [file peerj-11-15236-s004.zip › KO/CK-vs-T1_map/map05132.html]

KEGG PATHWAY: Salmonella infection - Reference pathway


|  |  |
| --- | --- |
| **Salmonella infection - Reference pathway** |  |

[
Pathway menu
| Organism menu
| Pathway entry
| Show description
| User data mapping
]

Salmonella infection usually presents as a self-limiting gastroenteritis or the more severe typhoid fever and bacteremia. The common disease-causing Salmonella species in human is a single species, Salmonella enterica, which has numerous serovars.
Following intestinal colonization Salmonella inject effector proteins into the host cells using a type III secretion system (T3SS), T3SS1. Then a small group of effector proteins induce rearrangement of the actin cytoskeleton resulting in membrane ruffles and rapid internalization of the bacteria.The T3SS2 is responsible for translocating effector proteins that direct Salmonella-containing vacuole (SCV) maturation. The majority of the bacteria are known to survive and replicate in SCV.

|  |  |  |
| --- | --- | --- |
| Reference pathway | 184% 150% 122% 100% 82% 67% 55% | 图片下载 |
